# Cancer-derived exosomal miR-197-3p confers angiogenesis via targeting TIMP2/3 in lung adenocarcinoma metastasis

**DOI:** 10.1038/s41419-022-05420-5

**Published:** 2022-12-09

**Authors:** Rui-Min Chang, Yao Fu, Jun Zeng, Xiao-Yan Zhu, Yang Gao

**Affiliations:** 1 Department of Thoracic Surgery, Xiangya Hospital, Central South University, Changsha, 410008 Hunan Province China; 2 Hunan Engineering Research Center for Pulmonary Nodules Precise Diagnosis & Treatment, Changsha, 410008 Hunan Province China; 3 National Clinical Research Center for Geriatric Disorders, Changsha, 410008 Hunan Province China; 4 Hunan Key Laboratory of Skin Cancer and Psoriasis, Changsha, 410008 Hunan Province China; 5 Department of Anesthesiology, Xiangya Hospital, Central South University, Changsha, Hunan Province China; 6 Xiangya Lung Cancer Center, Xiangya Hospital, Central South University, Changsha, 410008 Hunan Province China

**Keywords:** Cell migration, Non-small-cell lung cancer

## Abstract

Cancer-derived exosomal miRNAs are implicated in tumorigenesis and development of lung adenocarcinoma (LUAD). The objective of this study is to unravel the biological function of exosomal miR-197-3p in LUAD metastasis. qRT-PCR showed that elevated miR-197-3p in LUAD tissues was positively correlated with LUAD metastasis. CCK-8, tube formation, transwell and wound healing assays revealed that exosomal miR-197-3p from LUAD cells promoted the proliferation, angiogenesis and migration of HUVECs in vitro. LUAD cells-derived exosomal miR-197-3p also facilitated tumor growth and angiogenesis in LUAD cells-derived tumor xenograft model. TIMP2 and TIMP3 were identified as target genes of miR-197-3p in HUVECs by bioinformatics analysis and luciferase reporter assay. Functional studies illustrated that exosomal miR-197-3p promoted angiogenesis and migration via targeting TIMP2 and TIMP3 in HUVECs. In vivo data further supported that exosomal miR-197-3p promoted lung metastasis via TIMP2/3-mediated angiogenesis. In conclusion, LUAD cells-derived exosomal miR-197-3p conferred angiogenesis via targeting TIMP2/3 in LUAD metastasis.

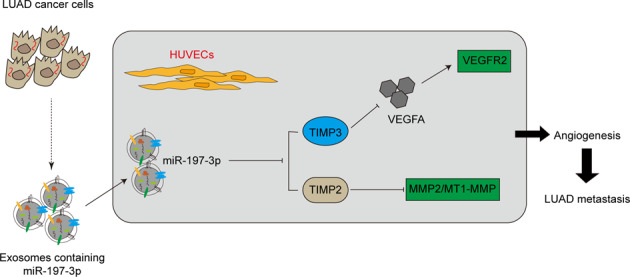

## Introduction

Lung cancer remains the leading cause of cancer mortality [1]. Lung adenocarcinoma (LUAD), a form of non-small cell lung cancer (NSCLC), accounts for around 40% of all lung cancers [2]. A majority of patients were frequently diagnosed with metastasis or in advanced stages, leading to limited therapeutic strategy and poor prognosis [3]. Metastasis is one of the major causes of high recurrence incidence and poor 5-year survival rate of LUAD. To improve the clinical outcomes, it is urgent to gain more insight into the molecular mechanism of LUAD metastasis.

Metastasis is a multi-step process in lung cancer, including tumor growth, cell migration, invasion, and angiogenesis. These processes occur in overlap or simultaneously, ultimately leading to distant metastasis [4]. To promote tumor growth, tumor cells activate angiogenic network for oxygen and nutrient supply. Considerable attention has been focused on angiogenesis, the new blood vessel formation from pre-existing vessels in tumors. It is generally accepted that vascular endothelial growth factors (VEGFs) and VEGF receptors (VEGFRs) play crucial roles in angiogenesis. In particular, VEGFA/VEGFR2 signaling is implicated in angiogenesis [5, 6]. In NSCLC, elevated VEGFR2 is involved in cancer progression and metastasis, and associated with poor prognosis [7, 8].

Communication between tumor cells and tumor microenvironment (TME) is critical for cancer development and progression. TME has effects on tumor cells by its composition, cell proportion, and activation state [9]. The cell type and activation state of TME stromal cells vary with cancer type and organ, as well as with environmental condition [9, 10]. Therefore, TME contributes to cancer progression via dynamic crosstalk. In recent years, the importance of exosomes in communication of tumor cells and TME has received increasing attention. Exosomes are small membrane vesicles between 30 and 100 nm in size which are secreted in biological fluid [11]. They serve as a mediator between donor and recipient cells by transportation of proteins, mRNAs, microRNAs (miRNAs) and other non-coding RNAs [12]. Notably, cancer-derived exosomal miRNAs have been reported to promote angiogenesis, remodel TME, and enhance pre-metastatic niche formation [13–15]. However, the functions of LUAD cells-derived exosomes are still under investigation.

Herein, we demonstrated that miR-197-3p expression was upregulated in serum exosomes of LUAD patients and LUAD tissues. Exosomes transported miR-197-3p from LUAD cells to human umbilical vein endothelial cells (HUVECs), thereby enhancing proliferation, angiogenesis and migration of HUVECs. Exosomal miR-197-3p also promoted tumor growth and angiogenesis in vivo. TIMP2 and TIMP3 were identified as functional targets of miR-197-3p in HUVECs. Exosomal miR-197-3p/TIMP2/3 axis contributed to angiogenesis and metastasis in vitro and in vivo. Our findings suggest that circulating miR-197-3p may be a promising target for anti-angiogenic treatment of LUAD.

## Materials and methods

### Collection of clinical specimens

A cohort of 62 LUAD tumor tissues and adjacent normal counterparts were collected from LUAD patients (primary LUAD, *n* = 30; metastatic LUAD, *n* = 32) from 2016 to 2019 at Xiangya Hospital of Central South University (Changsha, China). The fresh whole-blood samples from LUAD patients (primary LUAD, *n* = 30; metastatic LUAD, *n* = 32) and healthy donors (*n* = 25) were also collected for exosome purification. Informed consents were obtained from all recruiters. This study was approved by the Ethics Committee of Xiangya Hospital of Central South University.

### Cell culture, transfection and treatment

Normal lung epithelial cells (NLEC), HUVECs, LUAD cell lines including SK-LU-1, H358, H1650, H2030, H1975, A549, HCC827, PC9, H2009 and H1299 cells were purchased from Cell Bank of Type Culture Collection, Chinses Academy of Science (Shanghai, China), without contamination with mycoplasma. Cells authenticated before use were cultured in DMEM containing 10% FBS (Gibco, Grand island, NY, USA) at 37 °C. For overexpression experiments, the coding sequence (CDS) of human TIMP2 or TIMP3 was cloned into pcDNA3.1 vector (Invitrogen). Cells were transfected with miR-197-3p mimics, inhibitor, pcDNA3.1-TIMP2, pcDNA3.1-TIMP3 or their corresponding negative controls using Lipofectamine 3000 (Invitrogen, Carlsbad, CA, USA). For in vivo study, the stable transfection of overexpression or inhibition of miR-197-3p in A549 cells was performed using lentivirus (GeneChem, Shanghai, China). Lenti-mCherry containing a miR-197-3p overexpressing sequence, a miR-197-3p inhibiting segment or their negative controls were purchased from GeneChem, namely mimics NC, miR-197-3p mimics, inhibitor NC and miR-197-3p inhibitor. For the exosome treatment, exosomes were isolated from conditioned medium of LUAD cells and incubated with HUVECs as previous described [16].

### Histopathological analyses

For hematoxylin and eosin (H&E) staining, the mouse lung tissues were fixed with 4% paraformaldehyde for 24 h. The paraffin-embedded tissues were cut in 5 µm thick sections. H&E staining was employed to assess the alterations in morphology in these tissues using an optical microscope, and different fields were subsequently photographed.

For immunohistochemistry (IHC) analysis, paraffin-embedded tumor and lung tissues were deparaffinized and rehydrated as described [17]. After antigen retrieval and blocking, sections were incubated with anti-CD31 (1:50, ab182981, Abcam, Cambridge, UK), anti-TIMP2 (1:200, ab180630, Abcam) or anti-TIMP3 (1:100, ab213063, Abcam) antibody at 4 °C overnight, followed by the incubation with secondary antibody. The immunoreactivity was visualized by using DAB substrate (Thermo Fisher Scientific).

### Exosome purification and characterization

Exosomes were purified from A549 or H1299 cells-derived conditioned medium as previously described [15]. In brief, A549 or H1299 cells were cultured in DMEM with 10% exosome-free FBS. Conditioned medium was collected after 48 h, and centrifuged at 1000 × *g* to remove cells. After filtration through 0.22 μm filter, exosomes were precipitated using ExoQuick TC Exosome Precipitation Solution (System Biosciences, Palo Alto, CA, USA). Exosomes were purified from serum of patients with LUAD using Total Exosome Isolation Kit (from serum) according to the manufacturer’s instructions (Invitrogen). For transmission electron microscopy (TEM), exosomes were fixed with 2% paraformaldehyde and absorbed onto formvar-coated grids (200 mesh). The grids were then stained with 2% uranyl acetate and allowed to air dry. The samples were observed using TEM (Hitachi H7500 TEM, Tokyo, Japan).

### Exosome uptake assay

Exosome uptake assay was performed using PKH26 Red Fluorescent Cell Linker Kit (Sigma-Aldrich, St Louis, MO, USA) according to the manufacturer’s instruction. PKH26-labeled exosomes (10 μg) were resuspended in 100 μL PBS, and incubated with HUVECs (1 × 10^5^). Cells were harvested at 24 h for immunofluorescence analysis. Images were acquired by an Olympus confocal microscope (Olympus, Tokyo, Japan).

### Cell Counting kit-8 (CCK-8) assay

Cell proliferation was monitored by using CCK-8 kit (Beyotime, Haimen, China). Briefly, HUVECs or LUAD cells (1 × 10^3^) were plated in 96-well plates at 24 h prior to exosome treatment. HUVECs were incubated with different exosomes derived from A549 or H1299 cells. At designated time points, CCK-8 solution was added into each well, and incubated at 37 °C for 1 h. Absorbance at 450 nm was measured using a microplate reader (Bio-Rad Laboratories, Hercules, CA, USA).

### Colony formation assay

A549 and H1299 cells were seeded into 60-mm dishes and treated with designated exosomes. After 14 days, cells were fixed and stained with crystal violet. The visible colonies were photographed and counted under a microscope (Olympus, Tokyo, Japan).

### Tube formation assay

In vitro angiogenesis was assessed by tube formation assay as previously described [18]. Briefly, HUVECs were seeded onto Matrigel (Corning, Corning, NY, USA)-coated 24-well plates (1 × 10^5^) and treated with indicated exosomes derived from LUAD cells. After 24 h, tubes were photographed using an inverted microscope (Olympus).

### 3D spheroid sprouting assay

3D spheroid sprouting assay was conducted as previously shown [19]. Briefly, HUVECs (1 × 10^5^) were incubated with PBS or exosomes (10 μg) for 24 h. Cells were then harvested and cultured in endothelial cell EGM-2 medium (Lonza, Walkersville, MD, USA) containing 20% methocel in 96-well suspension plates (Corning). Spheroids were formed at 37 °C overnight, and suspended with EGM-2 medium containing collagen type I (3 mg/mL, BD Biosciences). After 1.5 h incubation, DMEM medium containing 10% FBS was added into each well. After 48 h, sprouts were photographed using an Olympus confocal microscope (Olympus).

### Wound healing assay

HUVECs or LUAD cells were incubated with PBS or indicated exosomes (10 μg) for 24 h. Cells (1 × 10^5^) were then collected and seeded in 6-well plates. The scratch was generated using a sterile 200 μL pipette tip as described [20]. After rinse with PBS, cells were then cultured at 37 °C for 24 h. Images of the scratches were acquired using a microscope (Olympus) at designated time points after scratching. The percentage of the healed wound area was measured as a ratio of the occupied area to the total area using Image Olympus IX71 (Olympus).

### Transwell migration assay

HUVECs or LUAD cells were incubated with PBS or indicated exosomes (10 μg) for 24 h. Cells (1 × 10^5^) were then harvested and cultured in serum-free medium in 24-well transwell upper chambers (Corning) without Matrigel coating. The lower chambers were filled with complete medium. After 24 h, the migrated HUVECs were fixed with methanol and stained with 0.1% crystal violet solution (Sigma-Aldrich). The stained cells from five random visual fields were then counted using a light microscope.

### Dual luciferase reporter assay

The putative binding sites between miR-197-3p and TIMP2/TIMP3 3’UTR were predicted using three bioinformatics programs, namely StarBase (https://starbase.sysu.edu.cn/index.php), TargetScan (https://www.targetscan.org/vert_72/) and miRTarBase (https://mirtarbase.cuhk.edu.cn/~miRTarBase/miRTarBase_2022/php/index.php). Among these three tools, StarBase was used to predict the binding sites between miR-197-3p and 3’UTR of both TIMP2 and TIMP3. The 3’UTR of TIMP2 or TIMP3 were cloned into psiCHECK^TM^-2 vector (Promega, Madison, WI, USA). Co-transfection of TIMP2 or TIMP3 3’UTR construct and miR-197-3p mimics or mimics NC were conducted using Lipofectamine 3000 (Invitrogen). Luciferase activity was assessed using Dual Luciferase Reporter Assay System (Promega).

### RNA isolation and quantitative real-time PCR

Total RNA was isolated from cells, tissues or exosomes using Trizol reagent (Invitrogen) or SeraMix Exosome RNA Purification Kit (System Biosciences), respectively. Reverse transcription was conducted using PrimeScript RT Reagent Kit (TaKaRa, Dalian, China). Quantitative real-time PCR was conducted using SYBR Green PCR Master Mix (Applied Biosystems). The expression levels of genes were normalized to that of GAPDH or U6. Synthetic *Caenorhabditis elegans* miR-39 (cel-miR-39) was used as an exogenous control for detect gene expression in exosomes. Results were analyzed using 2^−ΔΔCT^ method. The primers used in this study were listed in Supplementary Table 1.

### Protein extraction and western blot

Protein lysates were prepared using RIPA lysis buffer (Thermo Fisher Scientific), and protein concentration was determined by BCA Protein Assay Kit (Thermo Fisher Scientific). Protein lysates were separated by SDS-PAGE and transferred onto PVDF membrane (Bio-Rad). After blocking, the blot was incubated with primary antibody at 4 °C overnight, followed by incubation with secondary antibody. Chemiluminescence signal was visualized using SuperSignal Pico PLUS chemiluminescent Substrate (Thermo Fisher Scientific). The following primary antibodies were used in this study: anti-GM130 (1:1000, #12480); anti-CD54 (1:1000, #4915); anti-Annexin V (1:1000, #8555); anti-CD9 (1:1000, #13174); anti-TIMP2 (1:1000, #5738); anti-TIMP3 (1:1000, #5673); anti-GAPDH (1:1000, #5174); anti-VEGFR2 (1:1000, #9698); anti-ERK (1:1000, #4695); anti-p-ERK (1:1000, #4370); anti-MMP2 (1:1000, #87809); anti-MT1-MMP (1:1000, #13130) antibodies were purchased from Cell Signaling technologies (Beverly, MA, USA).

### LUAD cells-derived tumor xenograft model

Male BALB/c nude mice (*n* = 8 per group, 6-week-old) were purchased from SJA Laboratory Animal Co. Ltd (Changsha, China). All protocols were approved by Xiangya Hospital of Central South University. A549 cells (5 × 10^6^) were subcutaneously injected into the flank of mice. Mice were randomly divided into five groups: PBS, mimics NC-Exo, miR-197-3p mimics-Exo, inhibitor NC-Exo and miR-197-3p inhibitor-Exo. After 12 days, exosomes (10 μg) were injected into the xenograft tumor every 4 days as previously described [21, 22]. PBS was used as a blank control. The investigator was blinded to the group allocation during the experiment. Tumor sizes were measured every 4 days and tumor volumes were calculated with the formula: volume = 0.5 × width^2^ × length. Mice were sacrificed, and tumors were weighted and subjected to IHC analysis after five injections.

### LUAD lung metastasis model

The function of miR-197-3p was examined in vivo using experimental model of lung cancer metastasis with male BALB/c nude mice (*n* = 8 per group, 6-week-old), which were randomly divided into four groups: mimics NC-Exo, miR-197-3p mimics-Exo, miR-197-3p mimics-Exo+TIMP2, and miR-197-3p mimics-Exo+TIMP3. For in vivo study, the CDS of TIMP2 or TIMP3 was cloned into pCDH-CMV-MCS-EF1-copGFP lentiviral vector (GeneChem). A549 cells (1 × 10^7^) pre-treated with exosomes or/and TIMP2/TIMP3 lentiviral particles were injected into the lateral tail vein into BALB/c nude mice for observation of lung metastasis. 4 weeks after injection, mice were anesthetized and lungs were dissected for subsequent analyses. All procedures have got the approval of Animal Use and Care Committee of Xiangya Hospital of Central South University. The investigator was blinded to the group allocation during the experiment.

### Statistical analysis

Statistical analyses were conducted using the SPSS 20.0 software. Kaplan–Meier method was utilized for analyzing the survival curves, which was then evaluated the significance using a log-rank test. All error bars are displayed as the mean ± standard deviation (SD) derived from three independent experiments. The data were normally distributed, and variance was similar among the groups that are being statistically compared. Statistical analysis was performed using two-tailed Student’s *t* test or one-way analysis of variance (ANOVA) followed by Tukey’s post hoc test. Pearson’s correlation analysis was used to assess the degree of linear relationship between miR-197-3p and CD31, TIMP2, or TIMP3. *P* < 0.05 was considered statistically significant.

## Results

### Upregulation of miR-197-3p in LUAD tissues and exosomes positively correlates with metastasis and angiogenesis

**Fig. 1:**
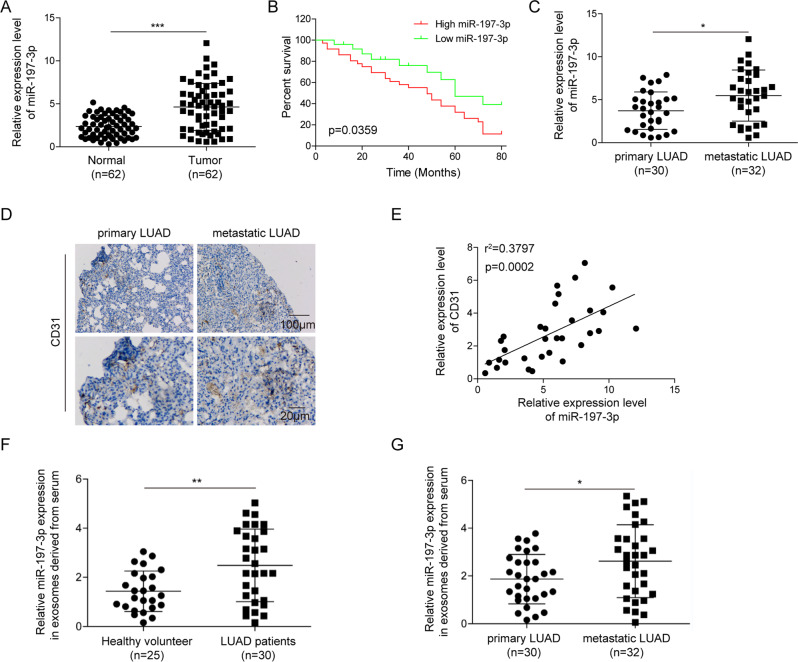
Upregulation of miR-197-3p in LUAD tissues and exosomes positively correlates with metastasis and angiogenesis. **A** The miR-197-3p level in normal and LUAD tissues was detected by qRT-PCR. **B** Kaplan–Meier survival analysis of LUAD patients. **C** The miR-197-3p level in primary and metastatic LUAD tissues was detected by qRT-PCR. **D** CD31 expression in primary and metastatic LUAD tissues was assessed by IHC. **E** Pearson correlation analysis between miR-197-3p and CD31 expression. **F** The exosomal miR-197-3p level from serum of healthy donor and LUAD patients was detected by qRT-PCR. **G** The exosomal miR-197-3p level from serum of primary and metastatic LUAD patients was detected by qRT-PCR. Data were representative images in **D**. *P < 0.05, **P < 0.01, ***P < 0.001.

Previous study demonstrated that miR-197-3p was highly expressed in LUAD tissues [23]. In order to investigate whether miR-197-3p is a metastasis-associated miRNA in LUAD, a series of experiments were carried out. As presented in Fig. 1A, miR-197-3p was significantly elevated in LUAD tissues, compared with paired adjacent normal lung tissues. Kaplan–Meier survival analysis also showed that overall survival was remarkably lower in LUAD patients with high miR-197-3p expression (Fig. 1B), indicating that high miR-197-3p level was associated with poor prognosis in LUAD. Moreover, qRT-PCR also revealed that miR-197-3p level in metastatic LUAD tissues was much higher than that in primary LUAD tissues (Fig. 1C). Given the important role of angiogenesis in cancer metastasis, we further examined the expression of vascular marker CD31 in both primary and metastatic LUAD tissues with different magnification. The expression of CD31 was significantly up-regulated in metastatic LUAD tissues in comparison with primary LUAD tissues as detected by IHC (Fig. 1D), suggesting that enhanced angiogenesis was observed in metastatic LUAD tissues. Pearson correlation analysis indicated that miR-197-3p and CD31 expression were positively correlated in metastatic LUAD (Fig. 1E). These data indicate that miR-197-3p in LUAD tissues positively correlates with metastasis and angiogenesis.

Emerging evidence shows the correlation between exosomal miRNAs and metastasis in various cancers [12]. We next examined the level of exosomal miR-197-3p in LUAD patients. Compared with that of healthy donor, miR-197-3p was dramatically increased in exosomes purified from the serum of LUAD patients (Fig. 1F). Moreover, miR-197-3p level in exosomes derived from serum of metastatic LUAD patients was significantly higher than that in primary LUAD patients (Fig. 1G). These data suggest that miR-197-3p is increased in exosomes derived from the serum of LUAD patients and associated with metastasis.

### Exosomes transport miR-197-3p from LUAD cells to HUVECs in vitro

**Fig. 2:**
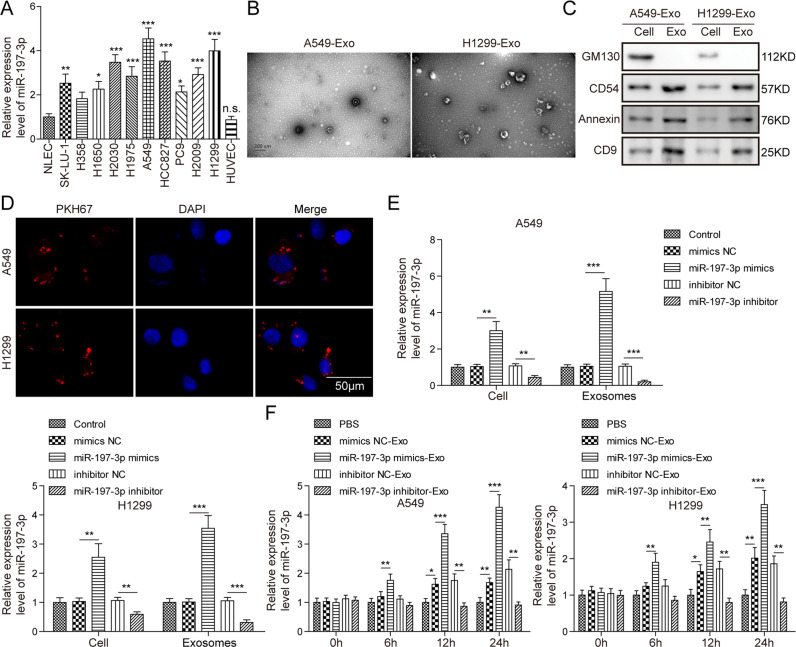
Exosomes transport miR-197-3p from LUAD cells to HUVECs in vitro. **A** The miR-197-3p level in NLEC, LUAD cells and HUVECs was detected by qRT-PCR. **B** The morphologies of A549 or H1299 cells-derived exosomes were observed by TEM. **C** Exosome markers and negative control GM130 were detected by western blot. **D** The uptake of PKH26-labeled exosomes was examined by confocal microscopy. Red: PKH26; Blue: DAPI. **E** Cellular and exosomal miR-197-3p levels in A549 and H1299 cells were detected by qRT-PCR. **F** HUVECs were treated with A549 or H1299 cells-derived exosomes for 0, 6, 12 and 24 h. MiR-197-3p level in HUVECs was detected by qRT-PCR. Data were representative images or were expressed as the mean ± SD of *n* = *3* experiments. *P < 0.05, **P < 0.01, ***P < 0.001.

To study the effects of exosomal miR-197-3p on umbilical vein endothelial cells, we investigated whether LUAD cells transported exosomal miR-197-3p to HUVECs. We first examined miR-197-3p levels in different LUAD cells by qRT-PCR. Compared with normal lung epithelial cells (NLEC) or HUVECs, miR-197-3p was highly expressed in LUAD cells, including SK-LU-1, H1650, H2030, H1975, A549, HCC827, PC9, H2009, and H1299 cells (Fig. 2A). A549 and H1299 cells with the relatively high miR-197-3p levels were selected for subsequent experiments. Exosomes purified from A549 or H1299 cells-derived conditioned medium were identified as membrane vesicles of 30–100 nm by TEM (Fig. 2B). In addition, these membrane vesicles were positive for exosome markers CD54, Annexin, and CD9, but negative for golgi apparatus marker GM130 which acted as a negative control (Fig. 2C). Cellular internalization of LUAD cells-derived exosomes into HUVECs was confirmed by exosome uptake assay in which PKH26-labeled exosomes were observed in HUVECs (Fig. 2D). qRT-PCR further revealed that transfection of miR-197-3p mimics or inhibitor in A549 or H1299 cells resulted in remarkable upregulation or downregulation of cellular and exosomal miR-197-3p levels, respectively (Fig. 2E). Furthermore, HUVECs were incubated with these exosomes for 0, 6, 12, and 24 h. As presented in Fig. 2F, miR-197-3p level in HUVECs exhibited a similar pattern with A549 or H1299 cells, and A549 or H1299 cells-derived exosomes regulated miR-197-3p level in HUVECs in a time-dependent manner. These findings confirm the important role of LUAD cells-derived exosomes in transporting miR-197-3p to HUVECs.

### Exosomal miR-197-3p promotes the proliferation, angiogenesis and migration of HUVECs

**Fig. 3:**
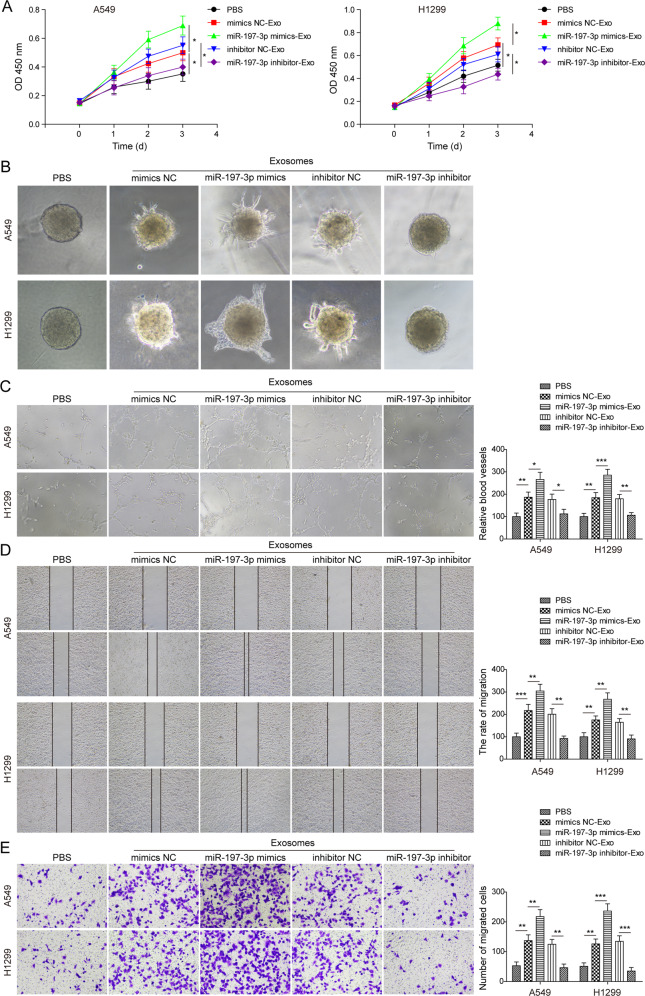
Exosomal miR-197-3p promotes proliferation, angiogenesis and migration in HUVECs. **A** Cell proliferation was monitored by CCK-8 assay. **B** Angiogenesis was measured by 3D spheroid sprouting assay. **C** Angiogenesis was assessed by tube formation assay with quantitative analysis (right panel). **D** Cell migration was assessed by wound healing assay with quantitative analysis (right panel). **E** Migration of HUVECs was monitored by transwell migration assay with quantitative analysis (right panel). Data were representative images or were expressed as the mean ± SD of *n* = 3 experiments. *P < 0.05, **P < 0.01, ***P < 0.001.

To further explore the function of exosomal miR-197-3p in HUVECs, we next assessed cell proliferation by CCK-8 assay. LUAD cells were transfected with mimics NC, miR-197-3p mimics, inhibitor NC or miR-197-3p inhibitor. Exosomes were then isolated from the conditioned medium of transfected LUAD cells, and HUVECs were subjected to designate treatment with A594 or H1299 cells-derived exosomes for 0, 24, 48, and 72 h. As shown in Fig. 3A, miR-197-3p-overexpressing exosomes (miR-197-3p mimics-Exo) dramatically promoted the proliferation of HUVECs in a time dependent manner, whereas miR-197-3p-knockdown exosomes (miR-197-3p inhibitor-Exo) exerted an opposite effect. Moreover, 3D spheroid sprouting (Fig. 3B) and tube formation (Fig. 3C) assays unequivocally revealed that miR-197-3p mimics-Exo promoted capillary-like tube formation of HUVECs, while miR-197-3p inhibitor-Exo inhibited the formation of tube-like structure, suggesting that A594 or H1299 cells-derived exosomal miR-197-3p was implicated in regulating angiogenesis. Additionally, wound healing (Fig. 3D) and transwell migration (Fig. 3E) assays further showed that miR-197-3p mimics-Exo or miR-197-3p inhibitor-Exo enhanced or impaired cell migration in HUVECs, respectively. Collectively, these data indicate that LUAD cells-derived exosomal miR-197-3p promotes the proliferation, angiogenesis, and migration of HUVECs. It is worth noting that exosomes derived from A549 or H1299 cells, including mimics NC-Exo and inhibitor NC-Exo, transferred miR-197-3p to HUVECs, thus enhancing the angiogenic and metastatic capabilities of HUVECs.

### Exosomal miR-197-3p promotes tumor growth and angiogenesis in vivo

**Fig. 4:**
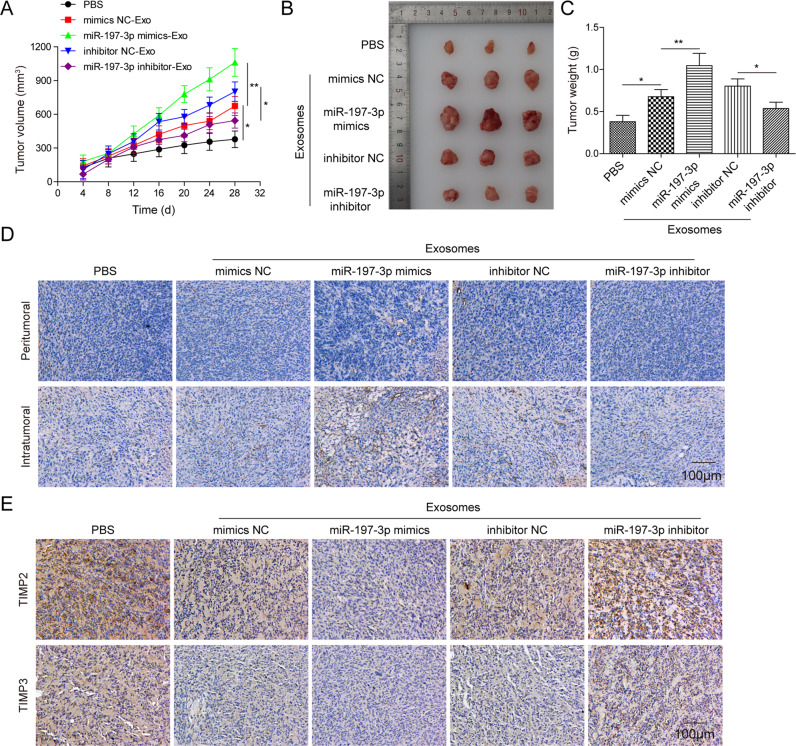
Exosomal miR-197-3p promotes tumor growth and angiogenesis in vivo. **A** Tumor volume was measured every 4 days. **B** Photographs of xenograft tumors. **C** Tumor weight was measured. **D** CD31 expression in tumor xenografts was assessed by IHC. **E** TIMP2 and TIMP3 expression in tumor xenografts were assessed by IHC. Data were representative images or were expressed as the mean ± SD of *n* = 3 experiments. *P < 0.05, **P < 0.01.

In order to validate the role of exosomal miR-197-3p in vivo, tumor xenograft model was established. A549 cells-xenograft bearing mice were subjected to designate treatment of exosomes every 4 days. As shown in Fig. 4A–C, miR-197-3p mimics-Exo led to significant induction of tumor volume and weight, whereas miR-197-3p inhibitor-Exo suppressed tumor growth, compared with corresponding controls. In addition, the vascular marker CD31 was predominantly expressed in tumor xenografts of miR-197-3p mimics-Exo group (Fig. 4D). In contrast, the immunoreactivity of CD31 was significantly repressed in tumor xenografts of miR-197-3p inhibitor-Exo group (Fig. 4D). IHC analysis also showed that miR-197-3p mimics-Exo remarkably decreased the expression of TIMP2/3 in the xenograft tumors, whereas miR-197-3p inhibitor-Exo exerted an opposite effect on TIMP2/3 expression (Fig. 4E). In addition, we further tested the effects of exosomes on the oncogenic properties of A549 and H1299 in vitro. As presented in Fig. S1A, B, CCK-8 and colony formation assays showed that no significant difference in cell proliferation was observed in miR-197-3p mimics-Exo or miR-197-3p inhibitor-Exo group, compared with corresponding controls. Similarly, the metastatic capacities of A549 and H1299 cells did not change after the treatment of different exosomes (Fig. S1C, D), indicating that exosomal miR-197-3p had no effect on the behavior of A549 and H1299 cells directly. Together, these results indicate that exosomal miR-197-3p promotes tumor growth and angiogenesis in vivo, and the effects observed on tumorigenicity and metastasis in the in vivo experiments are not due to a direct effect of exosomal miR-197-3p on tumor cells.

### TIMP2 and TIMP3 are functional targets of miR-197-3p in HUVECs

**Fig. 5:**
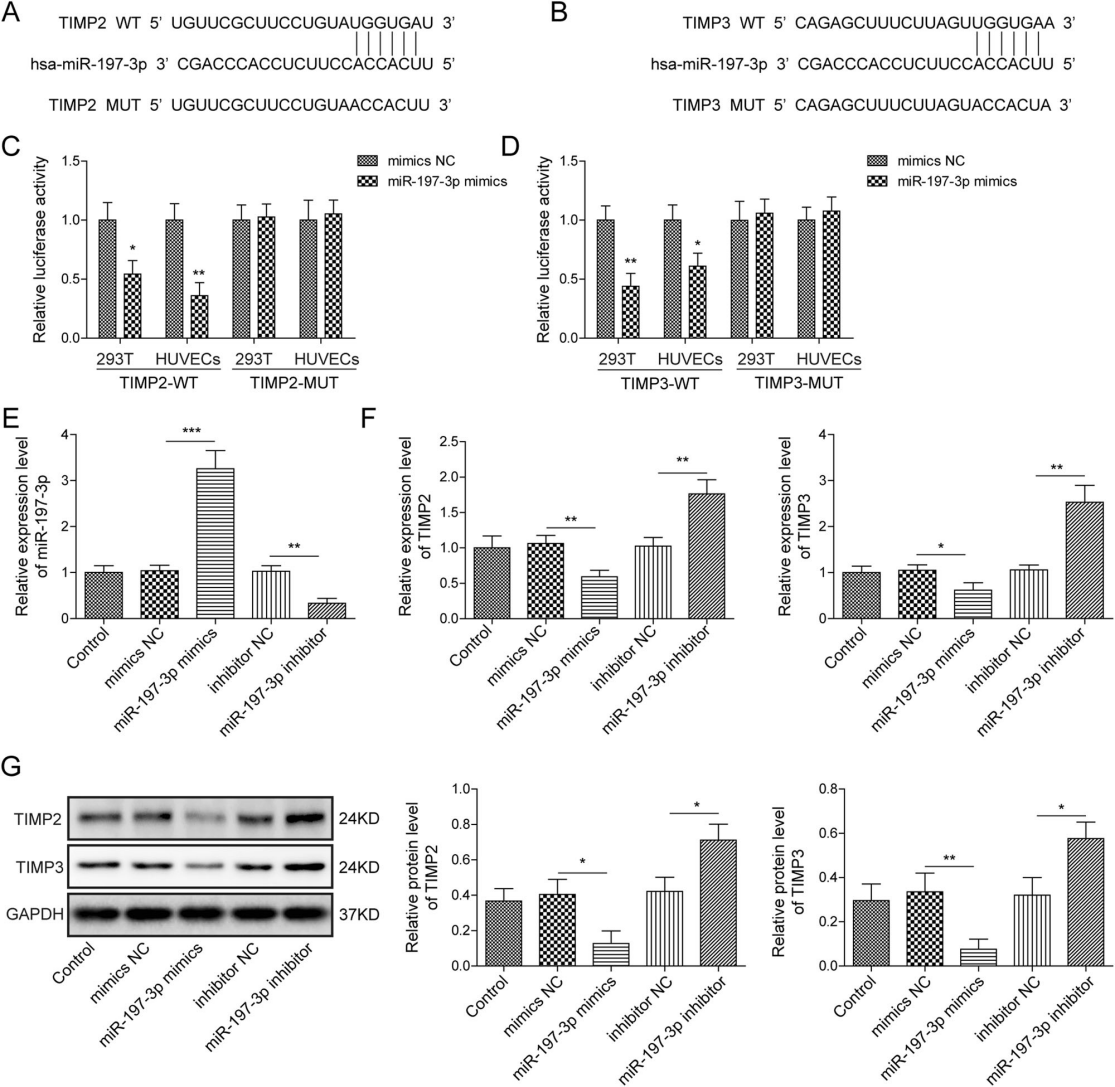
TIMP2 and TIMP3 are functional targets of miR-197-3p in HUVECs. **A** Putative binding sites between TIMP2 3’UTR and miR-197-3p. **B** Putative binding sites between TIMP3 3’UTR and miR-197-3p. **C, D** Relative luciferase activity was measured by dual luciferase reporter assay. **E** The miR-197-3p level in different HUVECs was determined by qRT-PCR. **F** The mRNA levels of TIMP2 and TIMP3 in different HUVECs were determined by qRT-PCR. **G** The protein levels of TIMP2 and TIMP3 in different HUVECs were determined by western blot. Data were representative images or were expressed as the mean ± SD of *n* = 3 experiments. *P < 0.05, **P < 0.01, ***P < 0.001.

We further explored the miR-197-3p target genes using bioinformatics programs. TIMP2 and TIMP3 were predicted as putative miR-197-3p target genes, and the putative binding sites between 3’UTR of TIMP2 or TIMP3 were shown in Fig. 5A, B, respectively. In both 293 T cells and HUVECs, co-transfection of wild-type 3’UTR of TIMP2 (TIMP2-WT) or TIMP3 (TIMP3-WT) cloned downstream of the luciferase gene and miR-197-3p mimics resulted in a dramatic reduction of luciferase activity, while the mutants of TIMP2 (TIMP2-MUT) or TIMP3 (TIMP3-MUT) abrogated this effect on luciferase activity (Fig. 5C, D). To further confirm the effect of miR-197-3p on TIMP2 and TIMP3 expression, gain- and loss-of function experiments were conducted. As expected, transfection of miR-197-3p mimics or inhibitor successfully increased to decreased miR-197-3p level in HUVECs (Fig. 5E), respectively. Additionally, miR-197-3p mimics or inhibitor negatively regulated TIMP2 and TIMP3 expression in HUVECs at both mRNA and protein levels (Fig. 5F, G).

**Fig. 6:**
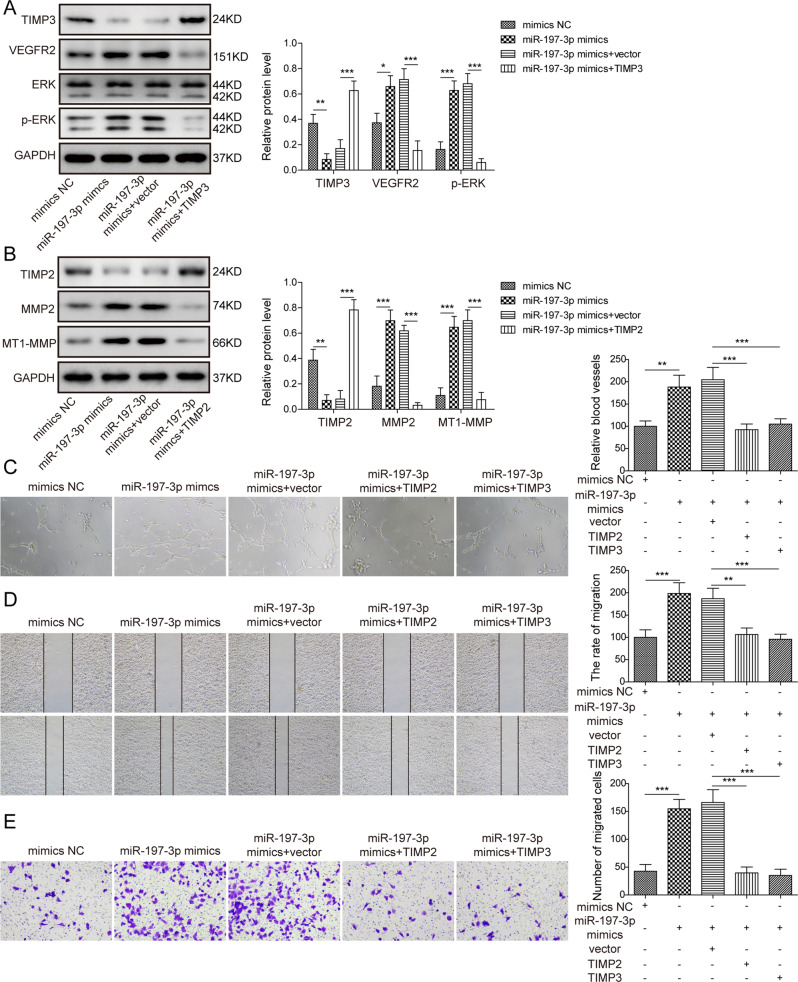
MiR-197-3p promotes angiogenesis and migration in vitro by targeting TIMP2/3 in HUVECs. **A** The protein levels of TIMP3, VEGFR2, ERK and p-ERK in different HUVECs were determined by western blot. **B** The protein levels of TIMP2, MMP2 and MT1-MMP in different HUVECs were determined by western blot. **C** Angiogenesis was assessed by tube formation assay with quantitative analysis (right panel). **D** Cell migration was assessed by wound healing assay with quantitative analysis (right panel). **E** Migration of HUVECs was monitored by transwell migration assay with quantitative analysis (right panel). Data were representative images or were expressed as the mean ± SD of *n* = 3 experiments. *P < 0.05, **P < 0.01, ***P < 0.001.

Moreover, we studied the downstream signaling and function of miR-197-3p/TIMP2 and miR-197-3p/TIMP3 axes in HUVECs. Western blot revealed that miR-197-3p mimics decreased TIMP3 expression, thereby inducing VEGFR2 expression and activating ERK signaling in HUVECs. These effects were reversed in miR-197-3p mimics+TIMP3 group, compared with control group (Fig. 6A). Interestingly, we also found that miR-197-3p mimics reduced TIMP2 expression, thus increasing MMP2 and MT1-MMP expression, whereas co-transfection of miR-197-3p mimics and TIMP2 remarkably attenuated these effects in HUVECs (Fig. 6B). Consistently, tube formation assay showed that miR-197-3p mimics promoted the formation of capillary-like tube, while overexpression of TIMP2 or TIMP3 abrogated the effect of miR-197-3p on in vitro tube formation (Fig. 6C). Wound healing assay coupled with transwell migration assay revealed that miR-197-3p mimics-enhanced migration of HUVECs was significantly attenuated in miR-197-3p mimics+TIMP2 or miR-197-3p mimics+TIMP3 group (Fig. 6D, E). Taken together, these data suggest that TIMP2 and TIMP3 are functional targets of miR-197-3p in HUVECs.

### Exosomal miR-197-3p promotes angiogenesis and migration via silencing TIMP2 and TIMP3 in HUVECs

**Fig. 7:**
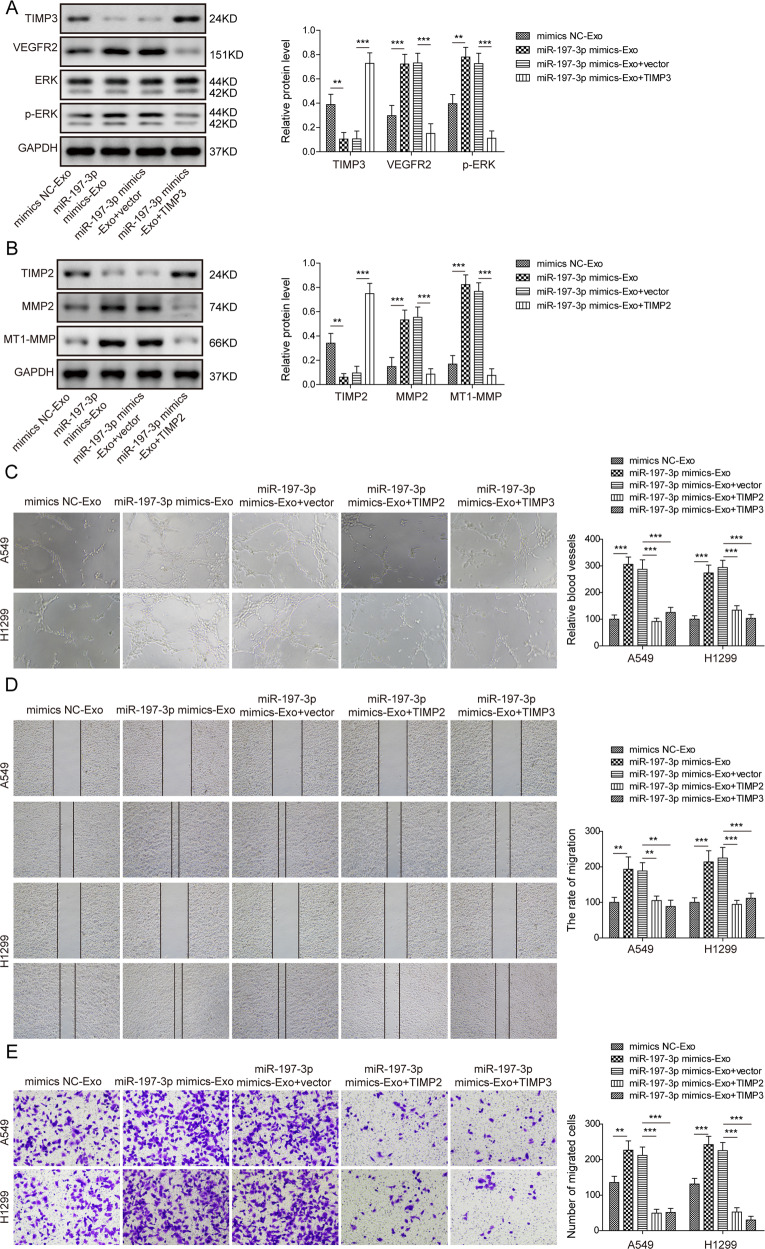
Exosomal miR-197-3p promotes angiogenesis and migration via silencing TIMP2/3 in HUVECs. **A** The protein levels of TIMP3, VEGFR2, ERK and p-ERK in different HUVECs were determined by western blot. **B** The protein levels of TIMP2, MMP2 and MT1-MMP in different HUVECs were determined by western blot. **C** Angiogenesis was assessed by tube formation assay with quantitative analysis (right panel). **D**, **E** Cell migration was assessed by wound healing and transwell assays with quantitative analysis (right panel). Data were representative images or were expressed as the mean ± SD of *n* = 3 experiments. **P < 0.01, ***P < 0.001.

To test whether A549 or H1299 cells-derived exosomal miR-197-3p exerted similar effects in HUVECs, exosomes were purified from conditioned medium of transfected A549 or H1299 cells. Consistently, miR-197-3p mimics-Exo-mediated changes of TIMP3 and VEGFR2, as well as ERK activation were attenuated by TIMP3 overexpression (Fig. 7A). Western blot also showed that miR-197-3p mimics-Exo-mediated changes of TIMP2, MMP2 and MT1-MMP expression were reversed by TIMP2 overexpression (Fig. 7B). In accordance with the data in Fig. 6, A549 or H1299 cells-derived exosomal miR-197-3p-enhanced tube formation and migration of HUVECs were abrogated by TIMP2 or TIMP3 overexpression (Fig. 7C–E). These results indicate that exosomal miR-197-3p promotes angiogenesis and migration via silencing TIMP2 and TIMP3 in HUVECs.

### Exosomal miR-197-3p promotes lung metastasis and angiogenesis in vivo by targeting TIMP2/3

**Fig. 8:**
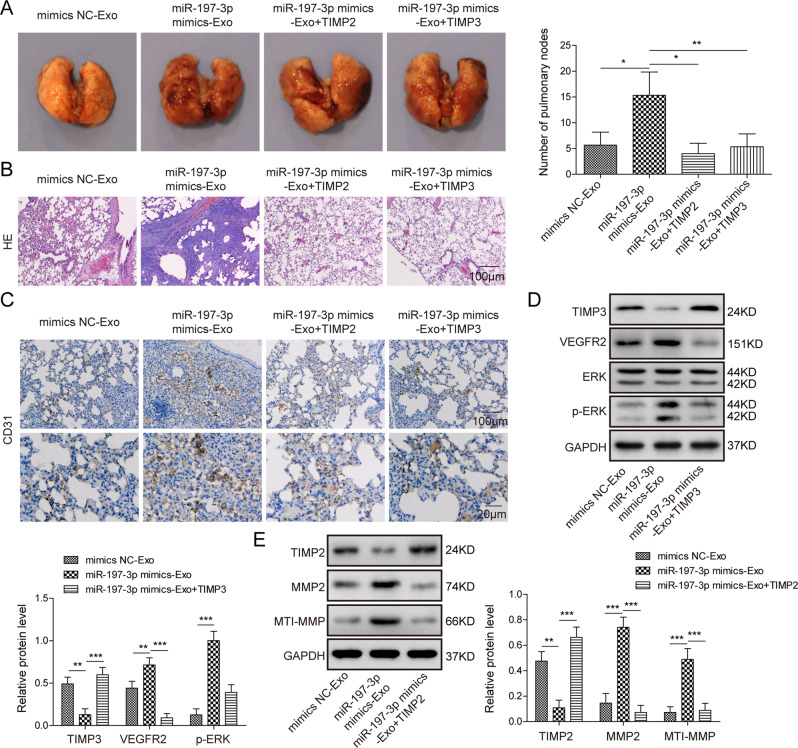
Exosomal miR-197-3p promotes lung metastasis and angiogenesis in vivo by targeting TIMP2/3. **A** Photographs and quantitative analysis of pulmonary metastatic nodules. **B** Histopathological changes of lung tissues were assessed by H&E staining. **C** CD31 expression in pulmonary metastatic nodules was assessed by IHC. **D** The protein levels of TIMP3, VEGFR2, ERK, and p-ERK in pulmonary metastatic nodules were determined by western blot. **E** The protein levels of TIMP2, MMP2, and MT1-MMP in pulmonary metastatic nodules were determined by western blot. Data were representative images or were expressed as the mean ± SD of *n* = 3 experiments. *P < 0.05, **P < 0.01, ***P < 0.001.

We next validated the effect of exosomal miR-197-3p on metastasis in LUAD lung metastasis model. The mice were injected with A549 cells after designate treatment of exosomes and TIMP2/3 lentiviral particles through tail vein. As shown in Fig. 8A, B, miR-197-3p mimics-Exo significantly increased the number of pulmonary metastatic nodules, while this effect was rescued by TIMP2 or TIMP3 overexpression. Consistent with these findings, IHC analysis showed that CD31 was markedly higher in pulmonary metastatic nodules of miR-197-3p mimics-Exo group (Fig. 8C). Nevertheless, overexpression of TIMP2 or TIMP3 further abolished miR-197-3p mimics-Exo-mediated induction of CD31 in pulmonary metastatic nodules (Fig. 8C). Moreover, downstream signaling of exosomal miR-197-3p/TIMP2 or miR-197-3p/TIMP3 axis was also examined in vivo. Similarly, miR-197-3p mimics-Exo-induced changes of TIMP3, VEGFR2 expression and ERK activation were blocked by TIMP3 overexpression (Fig. 8D). Overexpression of TIMP2 reversed the effects of miR-197-3p mimics-Exo on TIMP2, MMP2 and MT1-MMP expression in pulmonary metastatic nodules (Fig. 8E). Collectively, these data illustrate that LUAD cells-secreted exosomal miR-197-3p enhances lung metastasis via TIMP2/3-mediated angiogenesis in vivo.

### Serum exosomal miR-197-3p correlates with LUAD metastasis

Circulating exosomes extracted from serum of healthy donors and LUAD patients with or without metastasis were collected for assessing the level of miR-197-3p, and the results showed that exosomal miR-197-3p rose in the serum of LUAD patients and positively correlated with metastasis (Fig. S2A). We further analyzed the correlation between circulating miR-197-3p and miR-197-3p in LUAD tissues. Pearson correlation analysis illustrated that miR-197-3p in circulating exosomes and LUAD tissues were positively correlated (Fig. S2B). In addition, miR-197-3p was negatively correlated with TIMP2 or TIMP3 in metastatic LUAD tissues (Fig. S2C, D). These results might further illustrate that exosomal miR-197-3p is associated with LUAD metastasis, and negatively correlates with TIMP2 or TIMP3.

## Discussion

In this study, we demonstrated that miR-197-3p was elevated in both LUAD tissues and their serum exosomes, and the upregulation of miR-197-3p positively correlated with metastasis in LUAD patients. LUAD cells-derived exosomal miR-197-3p exerted oncogenic roles in vitro and in vivo. Mechanistic studies further revealed that exosomal miR-197-3p/TIMP2/3 axis was implicated in angiogenesis and metastasis in vitro and in vivo. Our findings shed novel insights on the biological role of exosomal miR-197-3p in LUAD.

Cancer-derived exosomes are involved in various biological processes, including tumor development and progression. Given the non-invasive properties of blood-based biomarkers, exosomes are recognized as promising diagnostic and prognostic biomarkers in lung cancer [24]. In addition to their importance in detection methods, the exchange of exosomal molecular compositions has also been documented. In particular, a number of studies have illustrated that miRNAs can be transferred by exosomes between cells, as well as between tumor cells and TME [25–27]. Over the last decade, an increasing attention has been focused on the function of miRNAs. As a class of non-coding RNAs, miRNAs with about 22 nt in length bind to the 3’UTR of target mRNAs, thereby regulating gene expression [28]. MiR-197-3p (also called miR-197) has been identified as an oncogenic miRNA in p53 wild-type NSCLC. Downregulation of miR-197-3p triggers p53-dependent cell apoptosis, and restrains tumor growth in vivo [29]. In clinical practice, miR-197-3p was found as a novel prognostic biomarker for NSCLC patients [30]. Moreover, several targets of miR-197-3p have been reported in lung cancer cells, including NOXA, Bcl-2-modifying factor (BMF) and lysine 63 deubiquitinase (CYLD) [23, 29]. However, the biological roles of exosomal miR-197-3p in LUAD have yet to be investigated. In line with the previous studies, we found that miR-197-3p was highly expressed in LUAD tissues [23], and the upregulation of miR-197-3p positively correlated with metastasis and angiogenesis. Interestingly, positive correlation was also observed between serum exosomal miR-197-3p level and LUAD metastasis. We thus hypothesized that exosomal miR-197-3p might play a pro-metastatic role in LUAD. Although miR-197-3p plays an oncogenic role in lung cancer cells, our findings suggested that exosomal miR-197-3p had no significant effect on the cell proliferative and metastatic properties of A549 and H1299 cells in vitro, indicating that the oncogenic effects of exosomal miR-197-3p in vivo are not attributed to its direct effects on lung cancer cells.

Angiogenesis plays an essential role in tumor progression and metastasis. Intratumoral hypoxia induces angiogenesis by modulating pro- and anti-angiogenic factors. It is well established that the pro-angiogenic factor VEGFA and its receptor VEGFR2 are principally responsible for tumor angiogenesis [6, 31, 32]. VEGFA binding to VEGFR2 initiates downstream signaling cascades, resulting in endothelial cell proliferation, migration and the formation of new blood vessels [33]. Anti-angiogenic agents in NSCLC fall into two categories: monoclonal antibodies and small molecule tyrosine kinase inhibitors. For instance, the VEGFA antibody bevacizumab which blocks VEGF-VEGFR binding is approved for use in NSCLC [31, 32]. Despite the success of anti-angiogenic agents, drug resistance and non-negligible toxicity remain the major concern. Therefore, novel anti-angiogenic targets gain increasing attentions recently. In the present study, LUAD cells-derived exosomal miR-197-3p was identified as a pro-angiogenic factor in vitro and in vivo. Elevated exosomal miR-197-3p in serum promoted metastasis via enhancing angiogenesis of recipient endothelial cells. Circulating exosomal miR-197-3p is a potential biomarker for prognosis in LUAD.

To further delineate the mechanism by which exosomal miR-197-3p regulated angiogenesis in LUAD, mechanistic studies were conducted. Since the known target genes of miR-197-3p are responsible for cell apoptosis in NSCLC [23, 29], we thus sought to identify the novel putative target genes of miR-197-3p responsible for angiogenesis. Given the anti-angiogenic roles of TIMP2 and TIMP3 [34, 35], these two predicted to be targeted by miR-197-3p attracted our attentions. Previous study illustrated that knockdown of TIMP3 enhanced tube formation in HUVECs [36]. In addition, knockout study revealed that loss of TIMP2 promoted angiogenesis, thereby accelerating osteoarthritis [37], confirming the anti-angiogenic role of TIMP2/3. TIMP family is composed of four members (TIMP1-4). They are originally identified as inhibitors of matrix metalloproteinases (MMPs), and involved in diverse biological processes, including cell proliferation, apoptosis, migration, invasion and angiogenesis [38, 39]. More importantly, TIMP2 and TIMP3 have been identified as novel biomarkers for cancers [39–41]. Notably, TIMP3 suppresses angiogenesis by blocking VEGF-VEGFR2 binding [34]. Consistently, we reported that exosomal miR-197-3p-mediated downregulation of TIMP3 led to VEGFR2 induction, thereby activated downstream ERK signaling and promoted angiogenesis. On the other hand, we also illustrated that exosomal miR-197-3p-mediated downregulation of TIMP2 was accompanied by the increase of MMP2 and MT1-MMP, suggesting that exosomal miR-197-3p might also enhance angiogenesis via TIMP2-mediated suppression of MMP2 and MT1-MMP. This observation was consistent with a previous report in gallbladder cancer [42]. In addition, miR-197-3p-regulated TIMP2/TIMP3 expression was also found in the xenograft model.

In conclusion, LUAD cells-derived exosomal miR-197-3p conferred angiogenesis via targeting TIMP2/3 in LUAD metastasis. Therefore, exosomal miR-197-3p level might be a promising prognostic biomarker and therapeutic target for anti-angiogenic treatment of LUAD.

## Supplementary information

Fig. S1

Fig. S2

Supplementary figures and table

Original Data File

Re-aj-checklist

## Data Availability

All data generated or analysed during this study are included in this published article.
