# Supplementary figures and images for: Cancer-derived exosomal miR-197-3p confers angiogenesis via targeting TIMP2/3 in lung adenocarcinoma metastasis
Source: Cell Death Dis. 2022 Dec 9;13(12):1032. doi: 10.1038/s41419-022-05420-5 (PMC9734149; doi:10.1038/s41419-022-05420-5)

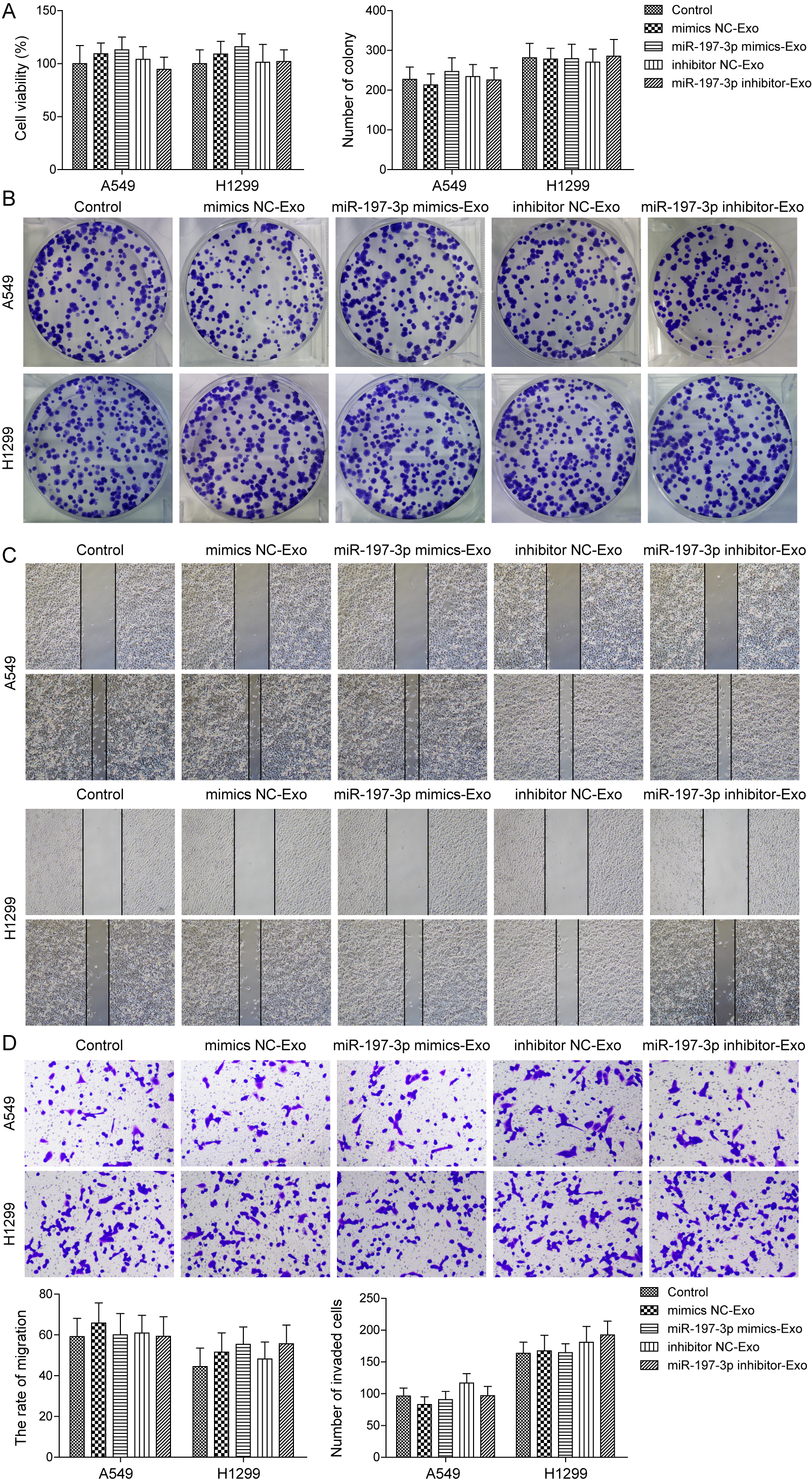

Supplement: Supplementary file 1 — Fig. S1 [file 41419_2022_5420_MOESM1_ESM.tif]

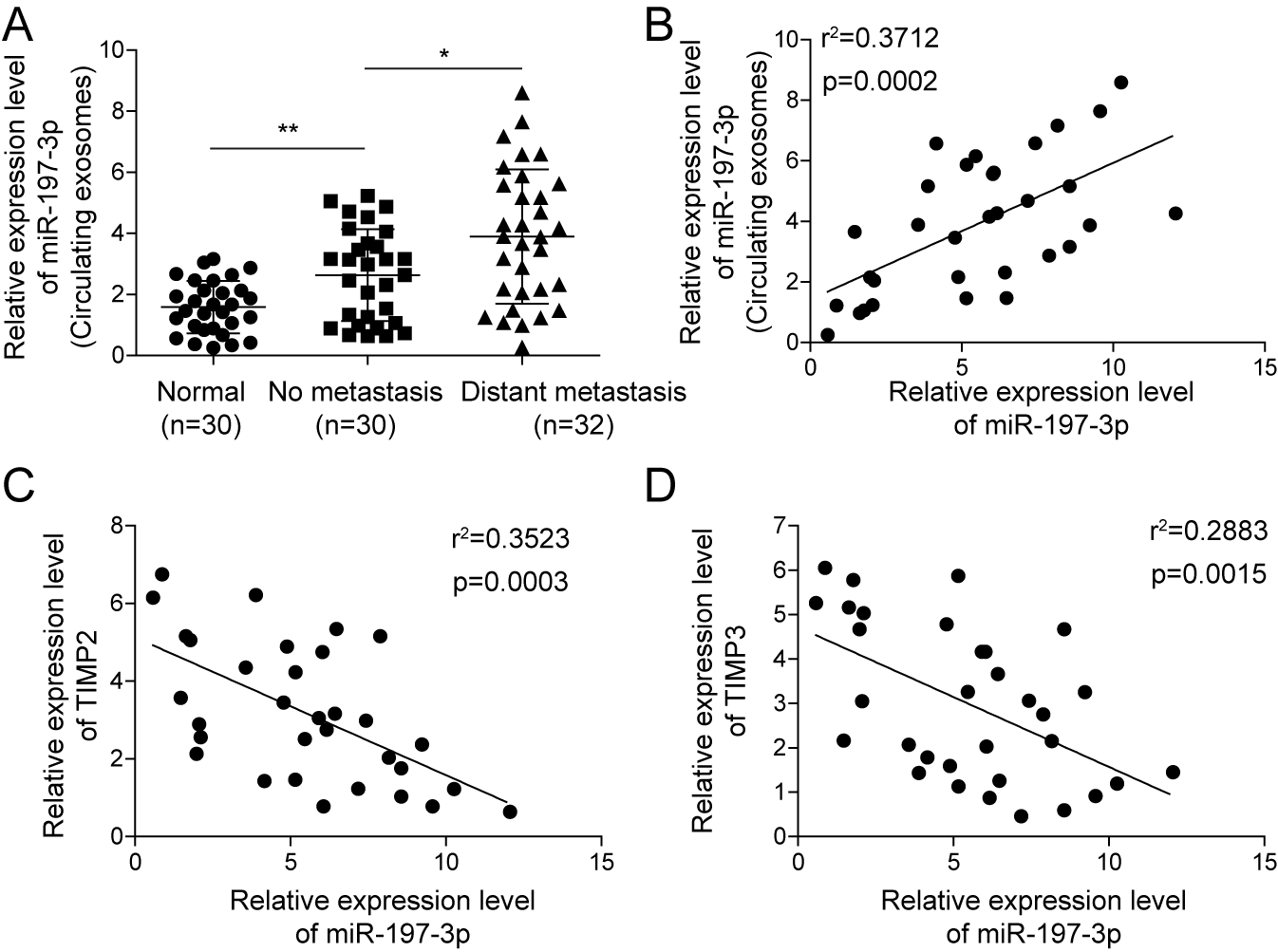

Supplement: Supplementary file 2 — Fig. S2 [file 41419_2022_5420_MOESM2_ESM.tif]

**Original western blots**


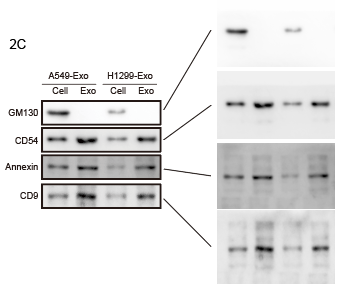

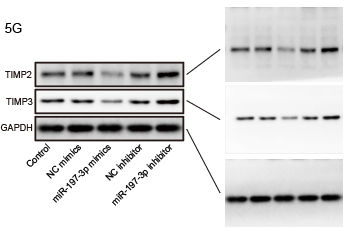

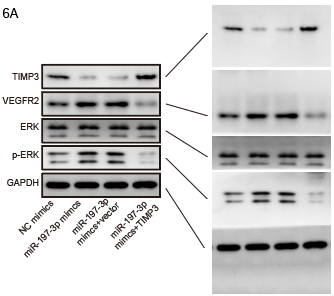

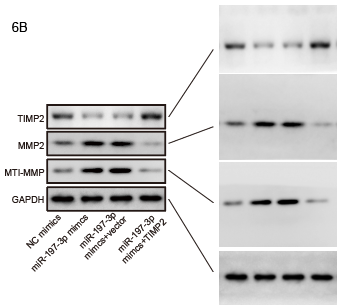

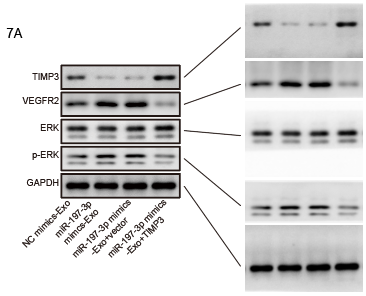

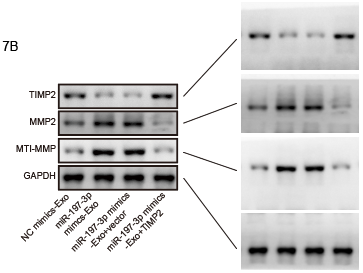

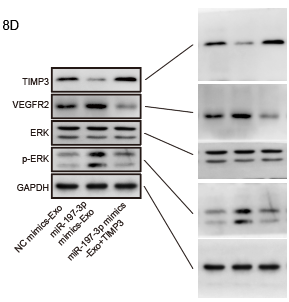

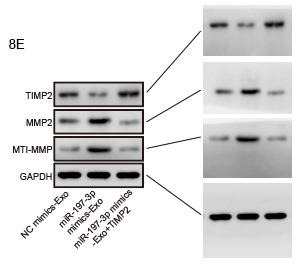

Supplement: Supplementary file 4 — Original Data File [file 41419_2022_5420_MOESM4_ESM.docx]
