# Supplementary material for: Cancer-derived exosomal miR-197-3p confers angiogenesis via targeting TIMP2/3 in lung adenocarcinoma metastasis
Source: Cell Death Dis. 2022 Dec 9;13(12):1032. doi: 10.1038/s41419-022-05420-5 (PMC9734149; doi:10.1038/s41419-022-05420-5)

**Supplementary figures**

**
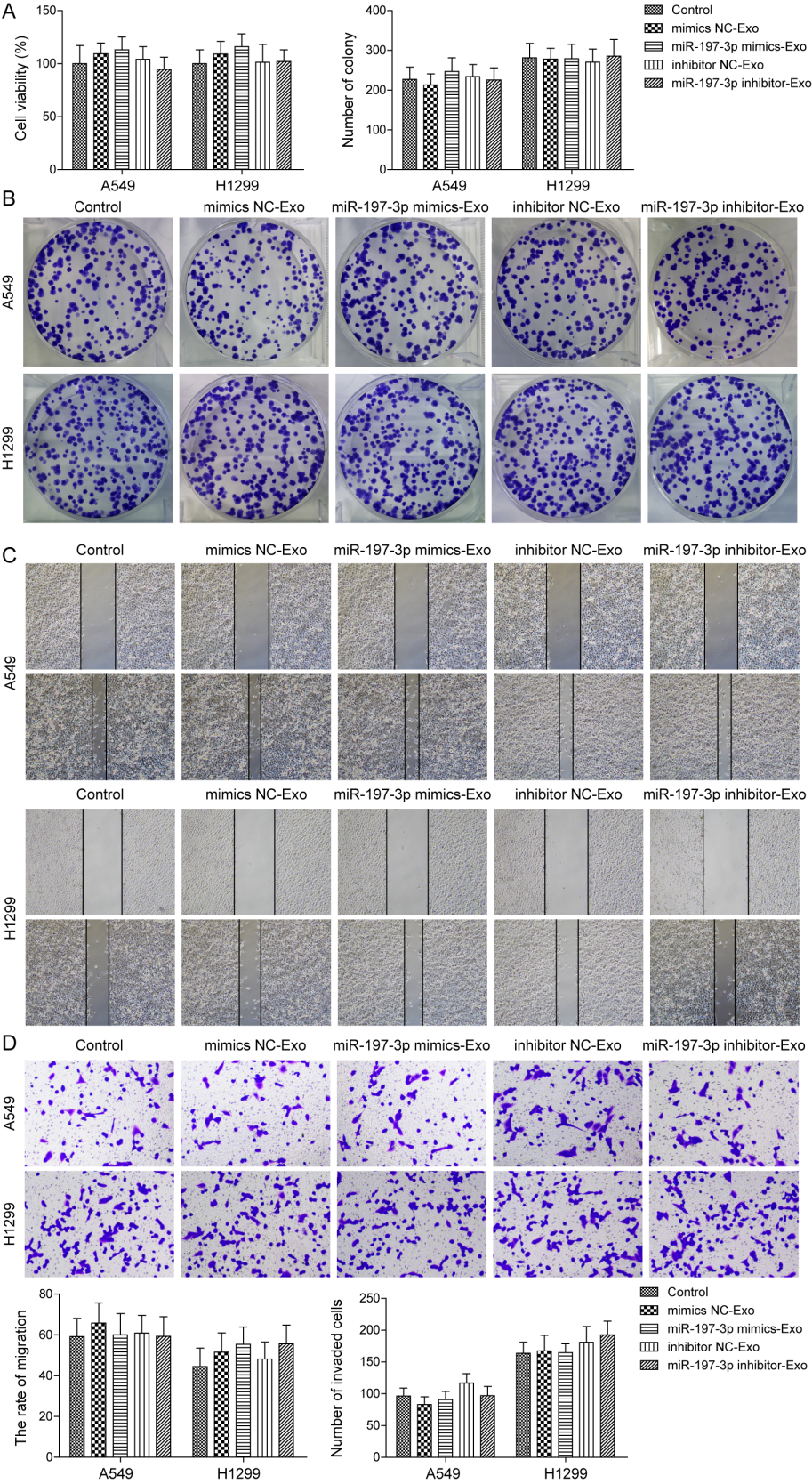
**

**Figure S1.** **Exosomal miR-197-3p has no effect on cell proliferation, migration and invasion in A549 and H1299 cells.**

1. Cell proliferation was monitored by CCK-8 assay. (B) Colony forming ability of cancer cells was assessed by colony formation assay. (C) Cell migration was assessed by wound healing assay. (D) Invasion of cancer cells was monitored by transwell invasion assay. Data were representative images or were expressed as the mean ± SD of *n = 3* experiments.

**
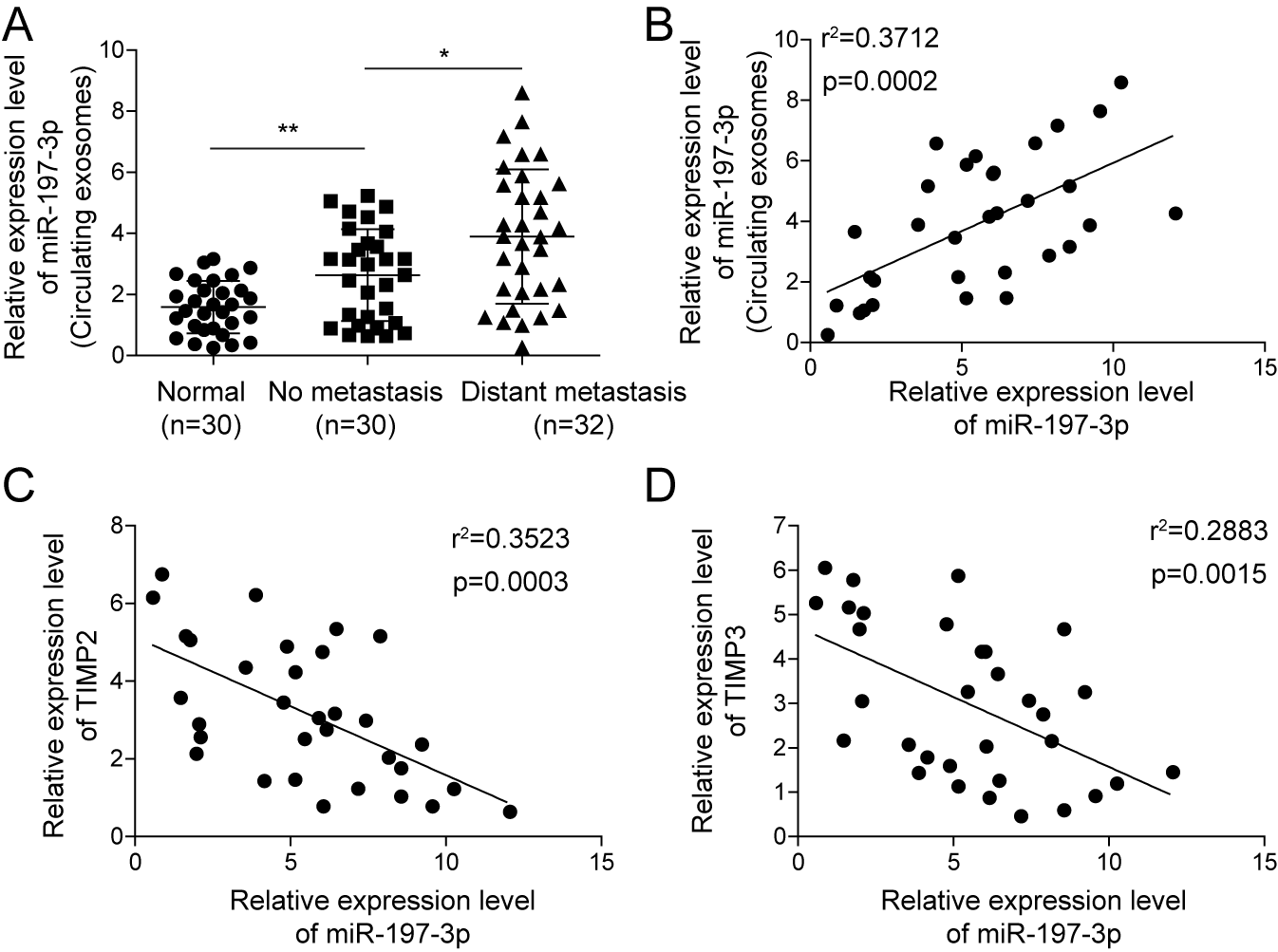
**

**Figure S2. Serum exosomal miR-197-3p correlates with LUAD metastasis.**

1. The miR-197-3p level from the serum of healthy donor, primary and metastatic LUAD patients was detected by qRT-PCR. (B) Pearson correlation analysis between exosomal miR-197-3p and miR-197-3p in LUAD tissues. (C, D) Pearson correlation analysis between miR-197-3p and TIMP2 or TIMP3 in metastatic LUAD tissues. *, *P* < 0.05, **, *P* < 0.01, ***, *P* < 0.001.

**Supplementary Table 1: The primers used in uantitative real-time PCR**

| **Primer** | **Sequence 5’-3’** |
| --- | --- |
| miR-197-3p forward | 5’-GCCTTCACCACCTTCTCCA-3’ |
| miR-197-3p reverse | 5’-AACGCTTCACGAATTTGCGT-3’ |
| TIMP2 forward | 5’-GATGCACATCACCCTCTGTG-3’ |
| TIMP2 reverse | 5’-GTGCCCGTTGATGTTCTTCT-3’ |
| TIMP3 forward | 5’-CTGACAGGTCGCGTCTATGA-3’ |
| TIMP3 reverse | 5’-AGTCACAAAGCAAGGCAGGT-3’ |
| U6 forward | 5’-CTCGCTTCGGCAGCACA-3’ |
| U6 reverse | 5’-AACGCTTCACGAATTTGCGT-3’ |
| GAPDH forward | 5’-CTGACTTCAACAGCGACACC-3’ |
| GAPDH reverse | 5’-GTGGTCCAGGGGTCTTACTC-3’ |

**Original western blots**


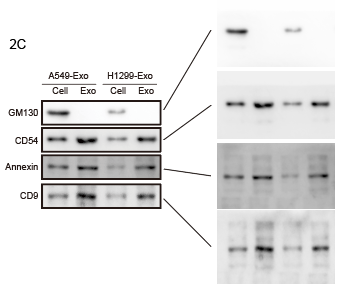

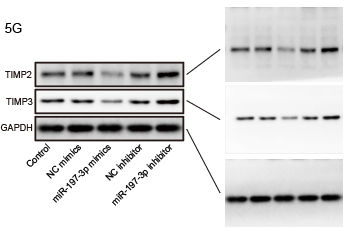

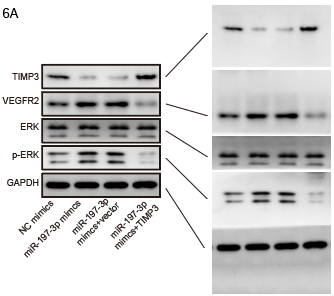

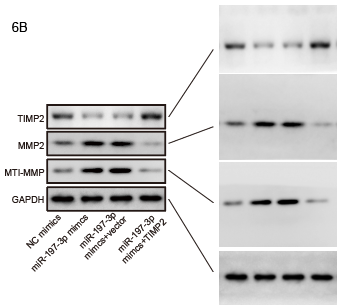

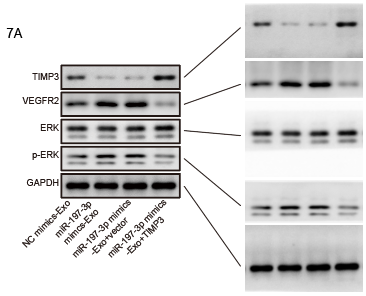

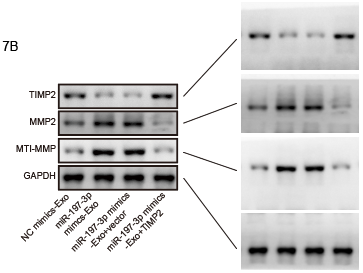

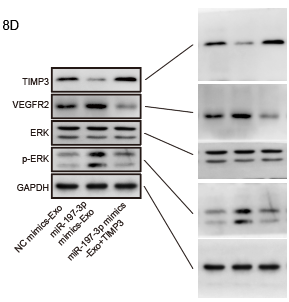

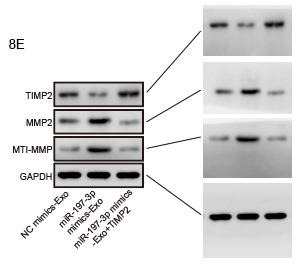

Supplement: Supplementary file 3 — Supplementary figures and table [file 41419_2022_5420_MOESM3_ESM.docx]
